# A feasibility study of immediate versus deferred antiretroviral therapy in children with HIV infection

**DOI:** 10.1186/1742-6405-5-24

**Published:** 2008-10-28

**Authors:** Jintanat Ananworanich, Pope Kosalaraksa, Umaporn Siangphoe, Chulapan Engchanil, Chitsanu Pancharoen, Pagakrong Lumbiganon, Jintana Intasan, Wichitra Apateerapong, Theshinee Chuenyam, Sasiwimol Ubolyam, Torsak Bunupuradah, Joep Lange, David A Cooper, Praphan Phanuphak, the HIV-NAT 010 Study Team

**Affiliations:** 1 The HIV Netherlands Australia Thailand Research Collaboration (HIV-NAT), the Thai Red Cross AIDS Research Center, Bangkok, Thailand; 2 The South East Asia Research Collaboration with Hawaii (SEARCH), Bangkok, Thailand; 3 Khon Kaen University, Khon Kaen, Thailand; 4 Chulalongkorn University, Bangkok, Thailand; 5 The International Antiviral Evaluation Center (IATEC), Amsterdam, the Netherlands; 6 The National Center for HIV Epidemiology and Clinical Research (NCHECR), University of New South Wales, Sydney, Australia

## Abstract

**Objective:**

To evaluate the feasibility of a large immediate versus deferred antiretroviral therapy (ART) study in children.

**Methods:**

We conducted an open-label pilot randomized clinical trial study in 43 Thai children with CD4 15 to 24% of starting generic AZT/3TC/NVP immediately (Arm 1) or deferring until CD4 < 15% or CDC C (Arm 2). Primary endpoints were recruitment rate, adherence to randomized treatment and retention in trial. Secondary endpoints were % with CDC C or CD4 < 15%. Children were in the trial until the last child reached 108 weeks. Intention to treat and on treatment analyses were performed.

**Results:**

Recruitment took 15 months. Twenty-six of 69 (37.7%) were not eligible due mainly to low CD4%. Twenty four and 19 were randomized to arms 1 and 2 respectively. All accepted the randomized arm; however, 3 in arm 1 stopped ART and 1 in arm 2 refused to start ART. Ten/19 (53%) in arm 2 started ART. At baseline, median age was 4.8 yrs, CDC A:B were 36:7, median CD4 was 19% and viral load was 4.8 log. All in arm 1 and 17/19 in arm 2 completed the study (median of 134 weeks). No one had AIDS or death. Four in immediate arm had tuberculosis. Once started on ART, deferred arm children achieved similar CD4 and viral load response as the immediate arm. Adverse events were similar between arms. The deferred arm had a 26% ART saving.

**Conclusion:**

Almost 40% of children were not eligible due mainly to low CD4% but adherence to randomized treatment and retention in trial were excellent. A larger study to evaluate when to start ART is feasible.

## Background

The Children with HIV Early Antiretroviral Therapy (CHER) study recently found that more infants randomized to deferring antiretroviral therapy (ART) until their CD4 fell below 25% died compared to those who started ART before the age of three months [1] but there is currently no randomized trial to guide when to start ART in children older than one year of age. Although, ART has significantly reduced HIV-related morbidity and mortality, it is associated with side effects, interference with daily activities and resistance [2-6]. Deferring the start of ART could possibly reduce these problems but the risk of HIV disease progression may increase. This randomized pilot study was conducted to explore the feasibility and HIV disease outcome of the immediate versus deferred ART strategy as a ground work for a larger study.

Recommendations of when to initiate ART differ between guidelines based on expert advice, published data of outcome in ART-untreated children and local resources [7,8]. Regular CD4 monitoring allows for opportunity to start ART prior to clinical progression as CD4 is the most important determinant for both short and long term HIV disease progression and death risks [7-9]. All guidelines recommend ART in children with severe HIV-related clinical events. Infants have a more rapid HIV disease progression, in fact, the efficacy and safety of immediate versus deferred ART strategy in older children is not known. Currently, in children age one year and up, the World Health Organization (WHO) guidelines for resource-limited countries and the Thai Ministry of Public Health guidelines recommend starting ART when CD4 is in the severe immune deficiency range according to age groups: 12–59 months (< 20%) and = > 5 years (< 15% or < 200 cells/mm^3^) [10-12]. The United States Guidelines recommend to start ART when CD4 is < 25% [13] while the Pediatric European Network for Treatment of AIDS recommend starting ART at CD4 < 20% in children ages 1 to 3 years and CD4 < 15% in older children [14].

We planned to enroll 43 children within one year and expected that many children would not be eligible as CD4 monitoring was not performed routinely and many children would have lower than required CD4. We also expected that more children would decline immediate ART as the Thai guidelines at that time recommended starting ART when CD4 was below 15%. Regarding the safety of the two treatment arms, we hypothesized that with close follow up and CD4 monitoring, ART can be deferred until CD4 < 15% in children ages 1 to 12 years old with Center for Disease Control and Prevention (CDC) clinical class A or B and CD4 15–24% without affecting HIV disease progression.

## Results

### Enrollment and retention rates

**Figure 1:**
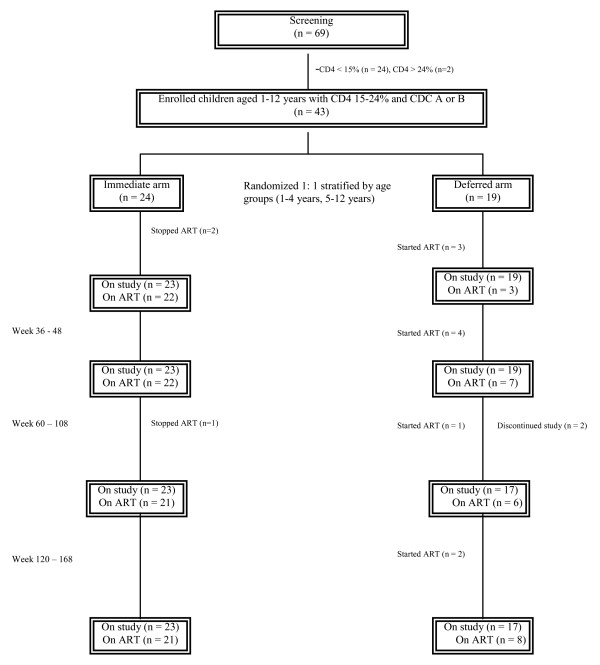
**Study design and patient disposition**. Footnote: All children in the deferred arm started ART because of protocol-defined CD4 criteria. All patients reached week 108. Some patients had additional follow up between weeks 120 to 168. Number of patients at last follow up visit in immediate/deferred arms = 21/15 at week 120, 13/11 at week 132, 8/5 week 144 and 7/4 at week 168). ART: antiretroviral therapy.

Between December 2001 and March 2003, 69 children were screened and 43 were enrolled at two sites (Figure 1). The recruitment began at the Bangkok site in December 2001, and in October 2002, the Khon Kaen site was included to increase recruitment. The overall recruitment rate was 4 children/month. The recruitment was more rapid towards the end of the study due to the inclusion of two sites. Twenty six (37.7%) failed screening due to the following reasons: 24 (92.3%) from CD4 < 15% and 2 (7.7%) from CD4 > 24% The children who were not eligible had a median age of 6.2 years (IQR 3.7 to 9.7) and median CD4 of 6.5% (IQR 3.3 – 10.8). All children accepted the randomized arm; however, three in the immediate arm stopped ART (one due to family's preference after the child had a mild nevirapine rash and two due to physician's recommendation after ART adherence cannot be ensured), and one deferred arm child was lost to follow up after the parents refused for the child to begin ART when the CD4 fell below 15%. Another deferred arm child was lost to follow up for unknown reason. The study ended when the last child reached week 108 of follow up with a median follow up time of 134 weeks, IQR 123 to 154, all in the immediate arm and 17 of 19 in the deferred arm completed the study. The median time spent on ART was 124 weeks (IQR 112–134) in the immediate arm children and 99 weeks (IQR 85–107) in the deferred arm children who initiated ART.

### Baseline characteristics

For the children who were enrolled, baseline characteristics are shown in Table 1. Forty-three children were randomized to immediate (n = 24) and deferred ART (n = 19). Overall, the median age was 4.8 years (IQR 2.7–6.6) with 17 males and 26 females. Most (84%) had CDC A clinical disease with a median CD4 of 19% (IQR 17–22) and CD4 count of 615 (541–824), and median viral load of 4.8 log_10 _copies/ml (IQR 4.3–5.3). Ninety and 77% of children had weight and height respectively in the normal ranges according to growth charts for Thai children (-1.5 to +2 standard deviation).

**Table 1:** Baseline characteristics

Characteristics	Study arms	
	Arm 1: Immediate (n = 24)	Arm2: Deferred (n = 19)

Gender [M : F], n (%)	10: 14 (42 : 58)	7: 12 (37 : 63)
Median age, years (IQR)	5.2 (2.4 – 8.0)	4.4 (2.7 – 5.8)
Age group, n (%)		
○ 1 – 4 yrs	12 (50)	10 (53)
○ 5 – 12 yrs	12 (50)	9 (47)
Median weight for age z-scores (WAZ) (IQR)	-1.0 (-1.5 to 0.4)	-0.1 (-1.5 to 0.3)
Median height for age z-scores (WAZ) (IQR)	-1.7 (-2.0 to -0.9)	-0.8 (-1.7 to 0.1)
HIV transmission, n (%)		
○ Blood product or transfusion recipient	2 (8)	0
○ Mother to child transmission	21 (88)	19 (100)
○ Unclear	1 (4)*	0
Exposed to AZT as prophylaxis against vertical HIV transmission, n (%)**	5/13 (39)	4/11 (37)
CDC categories, n (%)		
○ A	20 (83)	16 (84)
○ B	4 (17)	3 (16)
Median percent CD4+ count, % (IQR)	19 (16 – 22)	20 (17 – 22)
Median CD4+ count, cells/mm^3 ^(IQR)	649 (509 – 834)	615 (544 – 818)
Median plasma viral load, log_10 _copies/ml (IQR)	4.8 (4.3 – 5.3)	4.8 (4.1 – 5.1)
Median hemoglobin, g/dl (IQR)	10.8 (10.2 – 11.7)	11.2 (10.6 – 11.8)
Median alanine transferase, IU/L (IQR)	20 (11 – 30)	17 (15 – 23)
Median glucose (mg/dL) at week 48	83 (74 – 90)	81.5 (70.5 – 88)
Median cholesterol (mg/dL) at week 48	173 (153 – 195)	165 (150 – 186)
Median triglyceride (mg/dL) at week 48	69 (54 – 88)	98 (70 – 148)

*This child had no blood transfusion and his parents were not HIV-infected

**Information available in 24 patients

**Note: **all characters and baseline data did not differ between the two arms except median triglyceride was higher in the deferred arm (p 0.016).

### Initiation of ART in the deferred arm

Ten of 19 (53%) of deferred arm children started ART at a median time of 29 weeks (IQR 20 – 79), and a median CD4% and count of 12% (IQR 11–13, range 6 to 14) and 274 cells/mm^3 ^(IQR 220 to 394, range 137 to 555). The lowest CD4 drop to 6% (137 cells/mm^3^) was seen in a 31-month-old child at week 12 who had a baseline CD4 of 18% (571 cells/mm^3^). She did not have HIV disease progression and her CD4 rose after ART to 9% (402 cells/mm^3^) and 15% (505 cells/mm^3^) at weeks 24 and 36 respectively. Reason for ART initiation in the deferred arm was CD4 drop below 15% (n = 5) and CD4 drop by 25% (n = 5), and no difference in outcome was seen between these children.

### Clinical outcome

At the end of the study, no child had AIDS-defining illness or death. Four immediate arm children had new clinically diagnosed acid fast bacilli smear-negative pulmonary tuberculosis (TB) at a median time of 60 weeks (range 48 to 72), and a median CD4 of 28% (IQR 22 – 37) and 637 cells/mm^3 ^(IQR 568 – 894). They responded well to standard anti-TB treatment. The TB occurrence was not deemed related to immune reconstitution syndrome due to the large time gap between ART initiation and TB diagnosis and the high TB prevalence in the area. Weight for age Z score (WAZ) and height for age Z score (HAZ) were similar between arms.

### Immunological outcome

**Figure 2:**
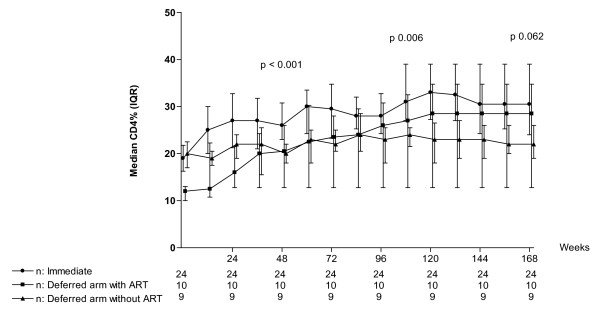
**Intention to Treat analysis of CD4% in the immediate arm, deferred arm without ART initiation and deferred arm with ART initiation**. * P-value represents difference within the three groups by by Kruskal Willis test.

Overall the immediate arm had a higher CD4% and CD4 count compared to the deferred arm (Table 2). At the end of the study, by ITT, no immediate arm child had CD4 < 15% and 3 children in the deferred arm had CD4 < 15%, all of whom had < 4 weeks of ART. In Figure 2, the CD4% in the immediate arm was higher than the deferred arm children who did not start ART (31%, IQR 24–39 versus 28.5%, IQR 12.8–34.8, p = 0.012). In deferred arm children who started ART, the median CD4% was 29%, IQR 13–35 and this was not statistically different than that of the immediate arm, p = 0.322.

**Table 2:** Outcomes at end of study (median time of 134 weeks)

**Data**	**Immediate** **n = 24**	**Defer** **n = 19**	**P**
HIV-related illness			
No. patient (%)	4 (16.7)	-	0.118
Adverse events			
No. patient (%)	22 (91.7)	19 (100)	0.495
No. event (% per month)	100 (13.3)	97 (17.0)	0.081
ARV-related adverse events			
No. patient (%)	7 (29.2)	4 (21.1)	0.728
No. event (% per month)	10 (1.3)	5 (0.7)	0.467
Median weight for age Z-scores (IQR)	-1.1 (-1.5 to -0.8)	-1.0 (-1.7 to 0.2)	0.525
Median height for age Z-scores (IQR)	-1.4 (-2.0 to -0.8)	-0.8 (-1.3 to -0.4)	0.171
Median CD4% (IQR)	31 (24 – 39)	23 (17 – 31)	0.032
Median CD4% change (IQR)	13.5 (4 – 18)	3 (-2 to 13)	0.012
Median viral load*, log (IQR)	1.7 (1.7 – 2.5)	3.1 (1.7 – 4.5)	0.039
Median change viral load, log (IQR)	-2.8 (-3.4 to -1.9)	-1.8 (-3.2 to -0.3)	0.079
Median Hemoglobin, g/dL (IQR)	11.7 (10.9 – 12.5)	11.7 (10.9 – 12.5)	0.961
Median alanine transferase, unit/mL (IQR)	15 (12.3 – 21.8)	18 (14 – 23)	0.418
Median triglyceride, mg/dL (IQR)	72 (46 – 109)	98 (61 – 156)	0.106
Median cholesterol, mg/dL (IQR)	158 (152 – 202)	160 (143 – 180)	0.197
Median glucose, mg/dL (IQR)	77 (67 – 87)	76.5 (72.3 – 90.5)	0.833

*Median viral load in the 10 deferred arm children who started ART was 1.7 log (IQR 1.7–1.7) which was similar to that in the immediate arm children (p = 0.58)

### Virological outcome

**Figure 3:**
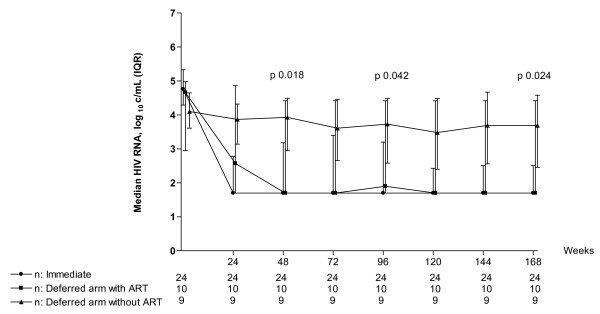
**Intention to Treat analysis of viral load in the immediate arm, deferred arm without ART initiation and deferred arm with ART initiation**. * P-value represents difference within the three groups by by Kruskal Willis test.

Overall the immediate arm had a lower viral load than the deferred arm (Table 2). By ITT, at the end of the study, the immediate arm had a lower median viral load of 1.7 log_10 _copies/ml (IQR 1.7 – 2.5) compared to the deferred arm children who did not start ART (median viral load 1.7 log_10 _copies/ml, IQR 1.7–4.8), p = 0.024 (Figure 3). However, once the deferred arm children initiated ART, their viral load was similar to the immediate arm (median viral load 1.7 log_10 _copies/ml, IQR 1.7 – 1.7), p = 0.580. The proportion of children with viral load < 400 and < 50 copies/ml were 75% (18 of 24) and 67% (16 of 24) in the immediate arm and 70% (7 of 10) and 60% (6 of 10) in the deferred arm children who initiated ART respectively, p = 1.000 and 0.714 respectively.

### Cost and toxicities

The deferred arm had 26% less time on ART with 31 months (IQR 28.1 – 33.4) and 25 months (IQR 21.2 – 26.7) of ART in the immediate and deferred arms respectively, p = 0.012. Median difference of time on ART between the two arms was 6.2 months (95% CI 3.3 – 9.9)

Both adverse events related and not related to ART occurred at a similar frequency between arms (Table 2). Anemia due to zidovudine (3 of 15 events) and rash due to nevirapine (3 of 15 events) accounted for most ART-related adverse events. One child switched from zidovudine to stavudine for anemia and 3 switched from nevirapine to efavirenz for rash. There were no differences between arms for hemoglobin, ALT, and fasting glucose, cholesterol and triglyceride (Table 2).

## Discussion

Our study showed that recruitment of ART-naïve children ages 1–12 years with CDC A or B and CD4 15–24% for a randomized trial of immediate versus deferred ART was feasible although the recruitment took longer than anticipated due to almost 40% of children being ineligible mainly from low CD4%. The randomized treatment was accepted in all families. Adherence to randomized treatment and retention rate in trial were high. No child had AIDS-defining illness or death. Four children in the immediate arm had clinically diagnosed pulmonary TB. Half of the deferred arm children started ART because of protocol-defined CD4 decline. For those who were on ART, the CD4 and viral load responses, and ART-related adverse events were similar regardless of treatment arms. The deferred arm had 26% less time on ART.

The risk for HIV disease progression rises with lower CD4%, higher viral load and younger age [7]. In earlier studies when no therapy or only zidovudine monotherapy was used, CD4 < 15% significantly increased the risk of AIDS and death [15-17]. Only one-third of untreated Thai children were alive without AIDS at 6 years of age [16]. Immediate versus deferred zidovudine monotherapy in children and adults have shown no difference in AIDS-free survival [18-21] but such randomized study in children older than one year of age using combination ART has not been done.

Our study was conducted when the government program providing free ART and monitoring was not available and the Thai Ministry of Public Health guidelines recommended ART initiation when CD4 was below 15%. Not surprisingly, almost 40% were ineligible from low CD4% since the majority of children had never had CD4 monitoring prior to the screening visit. The number of ineligible children can be reduced in subsequent studies if CD4 can be measured prior to screening as part of routine care. Improving recruitment rate by enrolling at multiple sites is important. We anticipated that some families of children randomized to immediate therapy would refuse the randomized arm, however, none did. The ample time spent during the consenting process likely alleviated parents' concerns about early therapy. One family in each arm subsequently refused the randomized treatment. This illustrates the importance of continued education about the study through out the trial.

None of the children had HIV disease progression despite the deferred arm children having lower CD4. This was likely due to the safety measures of frequent CD4 monitoring, immediate cotrimoxazole prophylaxis for *Pneumocystis jerovecii *pneumonia after the first CD4 < 15% and ART initiation shortly thereafter if repeated CD4 was confirmed to be low. Such safety measures are important when deferred ART is evaluated. It was not unexpected to find 4 children, all in the immediate arm, with pulmonary TB as Thailand is a high TB prevalence area.

The CD4 recovery and viral load suppression were similar in both arms despite the deferred arm having lower CD4 at ART initiation. Low baseline CD4 had been shown to dampen such responses [2,22,23]. We did not see more nevirapine hypersensitivity in the immediate arm despite higher CD4 being one of its risk factors [24]. The median difference of time on ART between the two arms in this small study was not large (6.2 months). Whether ART savings with deferred treatment will have a high impact in lowering cost burden for resource-limited countries, it needs to be evaluated in a larger study.

The main limitation for our study is the small sample size. We do not have the statistical power to show the differences in clinical efficacy and safety between the two treatment strategies. This sample size was; however, adequate for the purpose of evaluating feasibility of this strategy. The ART savings could be overridden by the costs for additional CD4 testing and monitoring visits which we were unable to quantify. We did not assess other important aspects that could affect the decision to start ART earlier or later such as neurocognitive function, vaccine response, quality of life and adherence to ART.

We learned from this pilot study about the feasibility and design of a more definitive randomized study addressing the question of when to start ART in children one year of age or older. Pre-screening CD4 as part of routine care, giving ample time for consenting, continuing education about the study through out the trial and including children at multiple sites are important for recruitment and adherence to treatment arms. The length of follow up and the sample size should be increased. With treatment becoming more effective and easier to take, the rates of AIDS and death may be low in both early and deferred ART; therefore, it is important to include minor HIV-related illnesses, non-HIV-related illnesses, growth, neurocognition and quality of life as outcomes.

## Conclusion

Our study suggests that it is feasible to evaluate the immediate versus deferred ART strategy in a larger study but many children were not eligible due to low CD4%. The adherence to randomized treatment and retention rate in trial were high. The strategy of deferred treatment appeared to be safe.

In fact, using the lessons learned from this pilot study, The PREDICT study (Pediatric Randomized to Early versus Deferred Antiretroviral Initiation in Cambodia and Thailand) conducted by our group has completed enrollment of 300 children in Thailand and in Cambodia. Results are expected in 2011.

## Methods

This open-label pilot randomized clinical trial was conducted between December 2001 and March 2005, Thai children with HIV at two sites in Thailand: The HIV Netherlands Australia Thailand Research Collaboration/Chulalongkorn University in Bangkok and Khon Kaen University in Northeast Thailand were recruited. After caregivers understood and signed informed consent form, children were screened for CD4% if they were 1–12 years old, had CDC clinical stage A or B and had never received ART other than zidovudine as part of PMTCT. The study was approved by the institutional review boards at Chulalongkorn and Khon Kaen Universities. All caregivers gave informed consent.

Based on published rates of CD4 rise, anticipation of excellent adherence and available funds [22], we enrolled 43 children in order to detect a difference in the proportion of children with CD4 < 15% at week 108 of 0% in the immediate arm and 30% in the deferred arm with 80 percent power (two-sided significance level of 5%) and 5% lost to follow up rate.

We based our study on the US guidelines HIV disease categorization [13], which HIV symptoms are categorized as none (CDC N), mild (CDC A), moderate (CDC B) and severe (CDC C) while the immunological status is categorized as normal (CD4 = > 25%), moderate immune suppression (CD4 15–24%) and severe immune suppression (CD4 < 15%)

At the time of this study, we did not include children younger than 1 year and those with CDC C or with CD4 < 15% as they would be at high risk of AIDS/death if randomized to deferring ART. We did not select children without symptoms or have normal CD4 as it was against the standard practice in Thailand at the time of this study to start ART in such children.

Patient disposition is shown in Figure 1. The children were randomized to either starting ART immediately (immediate arm, n = 24) or deferring ART until CD4 fell to < 15% in those with baseline CD4 20–24% or CD4 drop by 25% in those with baseline CD4 15–19% (deferred arm, n = 19). The basis for using CD4 drop by 25% was to avoid having children with CD4 15–19% starting ART soon after entering the study because of a minor CD4 drop. The randomization was stratified by age of 1–4 years old and 5–12 years old. In the deferred arm, a repeat CD4 was performed immediately if CD4 fell below ART initiation threshold. Cotrimoxazole was started immediately with the first CD4 fall below 15% and was continued for at least 3 months until two consecutive CD4 was above 15%. Standard doses according to the Thai guidelines of generic zidovudine/lamivudine/nevirapine provided by the Thai Government Pharmaceutical Organization were used. The drugs were given as individual drugs. All were available in pill and liquid forms except for nevirapine that was in tablet form only. A pill cutter was used to give the most accurate dose of nevirapine. Primary endpoints were 1) recruitment rate 2) adherence to randomized arm 3) retention in the study. Secondary endpoints were % children with CDC C or with CD4 < 15%, growth, median CD4%, median viral load, ART savings and ART-related adverse events. Children were followed monthly for the first 3 months and then every 3 months. CD4 by flow cytometry (BD Biosciences, Becton Dickenson and Company, San Jose, CA, USA), CBC, alanine transferase (ALT) were performed at every visit, and viral load (Roche Amplicor Ultrasensitive assay, Palo Alto, USA), and fasting lipids, glucose were performed every 24 weeks. The tests were performed on-site, both laboratories participated in the National Quality Assurance Program. The study ended when the last child reached week 108 of follow up. Adverse events were graded according to 1994 Adult and Pediatric Grading Tables of the Division of AIDS, National Institutes of Health, Bethesda, MD.

Intention-to-treat analysis (ITT) with last value carried forwards was performed for all endpoint comparisons between arms. On-treatment analysis (OT) was used to evaluate ART response. Data were censored at the last follow up visit for children who withdrew or were lost to follow up. Growth was assessed by WAZ and HAZ using Thai growth curves. Mann-Whitney test and Kruskal Willis test were used to compare continuous variables between these two and three groups. Chi-square and Fisher's exact test were used to test proportion differences. Chi-square test for trend was applied to identify whether there was a linear trend in the ordered proportion. In addition, we used McNemar test to test proportion differences in the same group.

The data management and analysis were conducted using SPSS for Windows, version 12 (SPSS Inc., Chicago, IL).

## Competing interests

The authors declare that they have no competing interests.

## Authors' contributions

JA designed the study, wrote the protocol, organized and coordinated the study, acquired the data and wrote the manuscript. PK coordinated the study at one site, acquired the data and helped write the manuscript. US analyzed the data and helped write the manuscript. CE, CP and PL were responsible for patients' inclusion and follow up, and acquiring the data. JI, WA and TC helped JA coordinate the study, acquire and monitor data at all sites. SU coordinated and defined strategies for analyzing all samples. TB assisted JA in following patients, acquiring data and writing manuscript. JL, DAC and PP helped JA design and write the protocol, and provided oversight and advice through out the study. All authors read and approved the final manuscript.
